# Risk factors for *in vivo* emergence of ceftazidime-avibactam resistance in KPC-producing *Klebsiella pneumoniae* and the associated resistance mechanisms: a case-control study

**DOI:** 10.1128/spectrum.00972-26

**Published:** 2026-05-04

**Authors:** Chien Chuang, Szu-Yu Liu, Yu-Chien Ho, Sheng-Hua Chou, Yi-Ru Huang, Wan Chin, Hsiang-Ling Ho, Liang Chen, Yi-Tsung Lin

**Affiliations:** 1 Division of Infectious Diseases, Department of Medicine, Taipei Veterans General Hospital 541403, Taipei, Taiwan; 2 School of Medicine, College of Medicine, National Yang Ming Chiao Tung University 210821, Taipei, Taiwan; 3 Department of Medicine, Kinmen Hospital, Ministry of Health and Welfare 371600, Kinmen, Taiwan; 4 Institute of Emergency and Critical Care Medicine, National Yang Ming Chiao Tung University 598162, Taipei, Taiwan; 5 Department of Pathology and Laboratory Medicine, Taipei Veterans General Hospital 46615, Taipei, Taiwan; 6 Department of Biotechnology and Laboratory Science in Medicine, National Yang Ming Chiao Tung University 548277, Taipei, Taiwan; 7 Department of Pharmacy Practice, School of Pharmacy and Pharmaceutical Sciences, University at Buffalo 15497, Buffalo, New York, USA; 8 Center for Infection Control, Taipei Veterans General Hospital 535217, Taipei, Taiwan; Emory University School of Medicine, Atlanta, Georgia, USA

**Keywords:** ceftazidime-avibactam, resistance, KPC, *Klebsiella pneumoniae*, risk factor

## Abstract

The emergence of ceftazidime-avibactam-resistant *Klebsiella pneumoniae* carbapenemase (KPC)-producing *Klebsiella pneumoniae* (CZA-R KPC-Kp), primarily due to KPC variants, poses a growing challenge to clinical practice. We aimed to investigate risk factors for *in vivo* development of CZA resistance in KPC-Kp and explore resistance mechanisms. A matched case-control study was conducted at Taipei Veterans General Hospital, including patients with sequential KPC-Kp isolates, between June 2019 and May 2025. Forty-five patients with CZA-R KPC-Kp and a prior CZA-susceptible KPC-Kp (i.e., cases) were matched 1:1 to controls with both the first and the second KPC-Kp isolates being CZA-susceptible, based on the interval between the first and second isolates. Logistic regression was used to determine risk factors for the emergence of CZA resistance. The median interval between the first and second isolates was 20 days. KPC variants accounted for 73.3% (33/45) of CZA-R KPC-Kp isolates, with 20 variant types identified. The most common KPC variant was KPC-33 (*n* = 11), followed by KPC-35 (*n* = 3). Most KPC variants-producing strains (81.8%, 27/33) exhibited restored imipenem susceptibility (minimum inhibitory concentration [MIC] ≤ 4 µg/mL). CZA use was an independent risk factor for the development of resistance (odds ratio [OR] = 9.47, *P* = 0.001), whereas carbapenem use was a protective factor (OR = 0.27, *P* = 0.015). In conclusion, CZA exposure was the only risk factor for CZA resistance development in KPC-Kp, whereas the use of carbapenem may reduce the risk, possibly due to restored imipenem susceptibility in most KPC variant-producing strains.

**IMPORTANCE:**

The emergence of ceftazidime-avibactam (CZA)-resistant *Klebsiella pneumoniae* carbapenemase (KPC)-producing *K. pneumoniae* poses a new threat to public health. Our study found that 73.3% of CZA-resistant strains carried KPC variants, and KPC-33 was the most frequent. Most KPC variants (82%) regained imipenem susceptibility. Exposure to CZA independently predicted resistance in KPC-Kp, whereas carbapenem use was protective. These findings highlight the importance of antibiotic stewardship and warrant further comparative study of meropenem-vaborbactam or imipenem-cilastatin-relebactam versus CZA for KPC-producing *K. pneumoniae* infections.

## INTRODUCTION

Carbapenemase-producing *Enterobacterales* (CRE) are listed as pathogens posing the greatest threat to public health due to limited treatment choices and high mortality (1). The predominant carbapenemases include *Klebsiella pneumoniae* carbapenemase (KPC) (Ambler class A), metallo-β-lactamases (Ambler class B), and OXA-48-like enzymes (Ambler class D), with geographic variability in their distribution across countries and regions (2). Among these, KPC is the most common mechanism mediating carbapenem resistance in *Enterobacterales* overall, particularly in the United States, several European countries, and Taiwan (2, 3).

Ceftazidime-avibactam (CZA) is recommended for the treatment of infections caused by KPC-producing *Enterobacterales* (4–6). However, with its increasing clinical use, CZA-resistant KPC-producing *Enterobacterales* have emerged (7). The primary mechanisms of CZA resistance involve mutations within the KPC enzyme, especially in the Ω-loop region (positions 164–179 of the KPC enzyme). Additional mechanisms include alterations in outer membrane porins OmpK35 and OmpK36, upregulation of the AcrAB efflux pump system, and increased copy numbers of *bla*_KPC_ (7). So far, more than 200 KPC variants have been reported worldwide (8). Notably, certain KPC variant-producing *Enterobacterales* strains have demonstrated restored susceptibility to carbapenems (9).

The emergence of CZA-resistant KPC-producing *K. pneumoniae* (CZA-R KPC-Kp) presents a growing clinical challenge due to high in-hospital mortality (22%–45.8%) and the lack of established optimal treatment regimens (10–12). CZA-R KPC-Kp may arise either through the *in vivo* evolution of resistance or via horizontal transmission between patients (13). However, factors associated with the *in vivo* development of CZA resistance remain poorly characterized, largely because of the limited number of CZA-R KPC-Kp strains (9–12). A recent review proposed that CZA treatment may be an independent risk factor for inducing mutations of *bla*_KPC_ (9). However, this hypothesis was not confirmed by a well-designed clinical study, such as a case-control study. Nonetheless, no studies have systematically evaluated whether additional clinical or microbiological factors contribute to the development of CZA resistance.

In this study, we aimed to investigate risk factors for *in vivo* emergence of CZA resistance in KPC-Kp. We also determined the KPC genotypes of these CZA-R KPC-Kp.

## MATERIALS AND METHODS

### Study design

This retrospective case-control study was conducted at Taipei Veterans General Hospital, a 2,900-bed tertiary-care teaching hospital in Taipei, Taiwan, between June 2019 and May 2025. Adults aged ≥18 years with at least two KPC-producing *K. pneumoniae* strains obtained from clinical specimens were included. Cases were defined as patients from whom a CZA-R KPC-Kp isolate was isolated 5 to 60 days following a CZA-S KPC-Kp isolate. Controls were defined as patients from whom a second CZA-S KPC-Kp isolate was recovered 5 to 60 days following a CZA-S KPC-Kp isolate. Each patient was included only once. Cases and controls were matched in a 1:1 ratio based on time at risk, defined as the interval between the two isolates (±2 days).

### Data collection

Patient demographics, antimicrobial agents used for more than 2 days between the first and second isolates, and clinical outcomes were collected from electronic medical records. Meropenem-vaborbactam (MVB), imipenem-cilastatin-relebactam (IMI/REL), and cefiderocol were not available in Taiwan during the study period. The definition of carbapenem use was imipenem, meropenem, and doripenem because all KPC-Kp strains, including wild-type KPC and KPC variant-producing *K. pneumoniae*, were resistant to ertapenem. Patients were considered immunocompromised if they had neutropenia, were receiving corticosteroid therapy (≥20 mg of prednisone or equivalent per day for ≥1 month), or were undergoing other forms of immunosuppressive therapy. Sequential organ failure assessment (SOFA) scores were calculated based on the day the first CZA-S KPC-Kp isolate was obtained. Source control was defined as the removal of necrotic tissue, removal of infected devices, or drainage of abscesses. Thirty-day mortality was defined as death occurring within 30 days following the isolation of the second KPC-Kp isolate.

### Microbiological data

Matrix-assisted laser desorption/ionization time-of-flight mass spectrometry (bioMérieux) was used to identify *K. pneumoniae* isolates, and the VITEK 2 system was used to determine the minimum inhibitory concentration (MIC) of antimicrobial agents. MIC of CZA was measured by Etest or the broth microdilution method. Results of susceptibility testing were interpreted based on the Clinical and Laboratory Standards Institute clinical breakpoints (14). CZA resistance was defined as an MIC ≥ 16 mg/L. Genes encoding capsular type and presence of carbapenemase (*bla*_KPC_, *bla*_IMP_, *bla*_NDM_, *bla*_VIM_, and *bla*_OXA-48_) were detected by polymerase chain reaction (PCR), followed by Sanger sequencing (15). All sequence types of KPC-Kp isolates were determined by using a multiplex PCR assay, as previously described (16).

Pulsed-field gel electrophoresis (PFGE) or whole-genome sequencing was performed to determine the clonal relatedness between the CZA-resistant strains and their susceptible parental strains. Only parts of the KPC-Kp strains underwent whole-genome sequencing due to resource limitations. The details of the PFGE protocol were described below: the bacteria were suspended in cell suspension buffer (100 mM Tris and 100 mM EDTA, pH 8.0) and adjusted to a turbidity of McFarland 4. Gel plugs were prepared by gently mixing 20 μL of Proteinase K (20 mg/mL stock, New England Biolabs), 400 μL of adjusted bacteria suspensions, and 400 μL SeaKem Gold (SKG) agarose (Lonza) in TE buffer (10 mM Tris and 1 mM EDTA, pH 8.0). The solidified gel plugs were digested in cell lysis buffer (50 mM Tris and 50 mM EDTA, pH 8.0, and 1% Sarcosine) containing proteinase K (0.5 mg/mL) and incubated at 56°C overnight. The plugs were then washed with sterile water and TE buffer at 56°C to remove proteinase K. Following digestion of plugs with the restriction endonuclease XbaI (New England Biolabs) at 37°C for 2 h, the plugs were loaded into 1% SKG agarose gel in 0.5× TBE buffer. Chromosomal macro-restriction fragment patterns were characterized by electrophoresis conditions that consisted of an initial switch time of 3 s and a final switch time of 35 s at an included angle of 120° and a gradient of 6 V/cm for 24 h with a linear ramp in 0.5× TBE at 14°C on a CHEF Mapper system (Bio-Rad Laboratories). Detailed protocol of whole-genome sequencing and core single-nucleotide polymorphism (SNP) analysis by snippy (https://github.com/tseemann/snippy) was described previously (17). Paired isolates with PFGE patterns differing by three or fewer restriction fragments or a difference of ≤21 SNPs were interpreted as closely related (18, 19).

### Statistical analyses

The chi-square or Fisher’s exact test was used to analyze categorical variables, whereas the two-tailed Student’s *t*-test was used to analyze continuous variables. To identify factors independently associated with the development of CZA resistance, multivariate logistic regression analysis was performed, and results were reported as odds ratios (ORs) and 95% confidence intervals (CIs). Variables with a *P* value of <0.1 in univariate analysis were included in the multivariate analysis. A *P* value <0.05 was considered statistically significant. Statistical analyses were conducted using SPSS version 26 (SPSS, Chicago, IL, USA).

## RESULTS

### Study population

During the study period, a total of 46 unique patients were identified from whom a CZA-R KPC-Kp isolate was recovered, with a CZA-S KPC-Kp counterpart obtained from the same patient 5–60 days earlier. Twenty pairs of CZA-R KPC-Kp and their parental CZA-S KPC-Kp strains (case-2, case-3, case-5, case-6, case-7, case-9, case-10, case-12, case-13, case-14, case-15, case-16, case-17, case-18, case-19, case-20, case-22, case-23, case-25, case-40) underwent core SNP analysis, which showed that the genetic distances between paired strains were less than 21 SNPs in all cases. Detailed strain numbers were listed in Table S1. The remaining 26 pairs of CZA-R KPC-Kp and their parent CZA-S KPC-Kp underwent PFGE. As illustrated in Fig. S1, 25 of 26 CZA-S and CZA-R KPC-Kp pairs exhibited indistinguishable or closely related PFGE patterns, differing by three or fewer bands. One pair (case-46) showed >3 different fragments, so it was excluded from further analysis.

In total, 45 cases were matched with 45 controls based on time at risk. The median interval between the first strain (CZA-S) and second strain (CZA-R) was 20 days (interquartile range, 14–32 days). The clinical characteristics were comparable between the case and control groups (Table 1). Detailed information regarding culture sites and intervals is presented in Table S2. Antibiotic exposures between the two time points are shown in Table 2.

**TABLE 1:** Demographic and clinical characteristics of the case and control groups^*a*^^,^^*b*^

Variable	Case (*n* = 45)	Control (*n* = 45)	*P* value
Age, years, median (IQR)	72 (59–82)	78 (65–85)	0.084
Male sex	29 (64.4%)	28 (62.2%)	0.827
Days between first and second KPC-Kp isolation, days, median (IQR)	20 (13–33)	20 (14–33)	0.939
ICU stay	21 (46.7%)	19 (42.2%)	0.671
*bla*_KPC_			0.110
KPC-2	39 (86.7%)	44 (97.8%)	
KPC-3	6 (13.3%)	1 (2.2%)	
Comorbidities			
Dementia	3 (6.7%)	4 (8.9%)	1.000
Diabetes mellitus	14 (31.1%)	19 (42.2%)	0.274
Hypertension	26 (57.8%)	26 (57.8%)	1.000
Coronary artery disease	10 (22.2%)	6 (13.3%)	0.270
COPD	5 (11.1%)	7 (15.6%)	0.535
Congestive heart failure	10 (22.2%)	13 (28.9%)	0.468
Cerebrovascular disease	5 (11.1%)	10 (22.2%)	0.157
Chronic kidney disease	25 (55.6%)	25 (55.6%)	1.000
Liver cirrhosis	3 (6.7%)	4 (8.9%)	1.000
Rheumatology disease	2 (4.4%)	2 (4.4%)	1.000
Malignancy			
Hematologic malignancy	3 (6.7%)	5 (11.1%)	0.714
Solid tumor	12 (26.7%)	12 (26.7%)	1.000
Immunocompromised state	17 (37.8%)	23 (51.1%)	0.203
Culture site			
First strain			
Respiratory	16 (35.6%)	16 (35.6%)	
Urine	9 (20.0%)	9 (20.0%)	
Blood	12 (26.7%)	15 (33.3%)	
Ascites/bile	6 (13.3%)	3 (6.7%)	
Wound	2 (4.4%)	2 (4.4%)	
Second strain			
Respiratory	23 (51.1%)	21 (46.7%)	
Urine	11 (24.4%)	5 (11.1%)	
Blood	3 (6.7%)	9 (20.0%)	
Ascites/bile	7 (15.6%)	6 (13.3%)	
Wound	1 (2.2%)	4 (8.9%)	
Charlson comorbidity index, median (IQR)	3 (2–5)	3 (2–6)	0.494
Invasive procedure between the first and second KPC-Kp strains			
Central venous catheter	30 (66.7%)	27 (60.0%)	0.512
Abdominal drain	12 (26.7%)	10 (22.2%)	0.624
Thoracic drain	5 (11.1%)	3 (6.7%)	0.714
Mechanically ventilated	24 (53.3%)	18 (40.0%)	0.205
Renal replacement therapy	18 (40.0%)	16 (35.6%)	0.664
Isolation of CR-GNB other than KPC-KP in the interval between the first and second KPC-Kp strains	20 (44.4%)	17 (37.8%)	0.520
SOFA score, median (IQR)	7 (4–12)	7 (3–10)	0.716
Source control	44 (97.8%)	44 (97.8%)	1.000
Clinical outcomes of second KPC-Kp strains			
30-day mortality	8 (17.8%)	15 (33.3%)	0.091
In-hospital mortality	14 (31.1%)	21 (46.7%)	0.130

^*a*^ Data are expressed as no. (%) unless otherwise specified.

^*b*^ IQR, interquartile range; ICU, intensive care unit; COPD, chronic obstructive pulmonary disease; CR-GNB, carbapenem-resistant Gram-negative bacteria; SOFA, sequential organ failure assessment.

**TABLE 2:** Antibiotic use in the interval between the first and second KPC-producing *Klebsiella pneumoniae* strains^*k*^

Antibiotic	Case (*n* = 45)	Control (*n* = 45)	*P* value
Ceftazidime-avibactam	42 (93.3%)	23 (51.1%)	<0.001
Days of ceftazidime-avibactam, median (IQR)	11 (8–14)	4 (0–9)	<0.001
First- and second-generation cephalosporin^*a*^	2 (4.4%)	2 (4.4%)	1.000
Third- and fourth-generation cephalosporin^*b*^	10 (22.2%)	15 (33.3%)	0.239
Cephamycin^*c*^	2 (4.4%)	3 (6.7%)	1.000
Penicillin^*d*^	14 (31.1%)	11 (24.4%)	0.480
Carbapenem^*e*^	9 (20.0%)	24 (53.3%)	0.001
Days of carbapenem, median (IQR)	0 (0–2)	3 (0–6)	0.001
Fluoroquinolone^*f*^	9 (20.0%)	14 (31.1%)	0.227
Aminoglycoside^*g*^	2 (4.4%)	7 (15.6%)	0.157
Colistin	7 (15.6%)	13 (28.9%)	0.128
Fosfomycin	3 (6.7%)	0 (0%)	0.242
Tigecycline	15 (33.3%)	21 (46.7%)	0.197
Minocycline	7 (15.6%)	3 (6.7%)	0.180
Anti-MRSA antibiotics^*h*^	28 (62.2%)	21 (46.7%)	0.138
Glycopeptide^*i*^	17 (37.8%)	14 (31.1%)	0.506
Linezolid	9 (20.0%)	7 (15.6%)	0.581
Daptomycin	6 (13.3%)	4 (8.9%)	0.502
Sulbactam^*j*^	4 (8.9%)	3 (6.7%)	1.000
Trimethoprim/sulfamethoxazole	1 (2.2%)	2 (4.4%)	1.000

^*a*^ Includes cefazolin and cefuroxime.

^*b*^ Includes ceftriaxone, ceftazidime, cefepime, cefixime, and cefoperazone/sulbactam.

^*c*^ Includes cefmetazole and flomoxef.

^*d*^ Includes amoxicillin/clavulanate, ampicillin/sulbactam, and piperacillin/tazobactam.

^*e*^ Includes imipenem/cilastatin, meropenem, and doripenem.

^*f*^ Includes ciprofloxacin, levofloxacin, and moxifloxacin.

^*g*^ Includes amikacin and gentamicin.

^*h*^ Includes vancomycin, teicoplanin, linezolid, and daptomycin.

^*i*^ Includes vancomycin and teicoplanin.

^*j*^ Includes sulbactam, ampicillin/sulbactam, and cefoperazone/sulbactam.

^*k*^ IQR, interquartile range; MRSA, methicillin-resistant *Staphylococcus aureus*.

Patients in the case group were more likely to have received CZA compared with those in the control group (93.3% versus 51.1%, *P* < 0.001), and the median duration of CZA therapy was also longer in the case group (11 days versus 4 days, *P* < 0.001). Detailed dosage and duration of CZA use are shown in Table S3. In contrast, patients in the case group were less likely to have received carbapenem (20.0% versus 53.3%, *P* = 0.001), and the median duration of carbapenem use was significantly shorter (0 day versus 3 days, *P* = 0.001). The carbapenems used in our study included meropenem and doripenem. In our study, only 2 of the 33 patients who received carbapenems were administered CZA concurrently, whereas the remaining 31 patients received carbapenem as an empirical antibiotic rather than in combination with CZA.

### Microbiological characteristics of KPC-Kp strains and CZA resistance mechanisms

All KPC-Kp strains evaluated in this study belonged to capsular type K47. Among the first KPC-Kp in both case and control groups, KPC-2 was the predominant KPC variant, accounting for 83 out of 90 strains (92.2%), while the remaining KPC-Kp strains were KPC-3-producing (7/90; 7.8%). In the case group, all paired CZA-S and CZA-R KPC-Kp strains belonged to ST11.

Among the subsequent 45 CZA-R KPC-Kp strains, the KPC genotypes had been documented for 34 strains in our previous study (12), including KPC-2, KPC-3, KPC-14, KPC-31, KPC-33, KPC-35, KPC-44, KPC-46, KPC-53, KPC-71, KPC-78, KPC-90, KPC-93, KPC-144, KPC-170, KPC-2&KPC-33 (co-harboring), and three distinct novel KPC variants.

A total of 33 strains (73.3%) producing KPC variants were identified from the 45 CZA-R KPC-Kp strains (Table S4), all of which were isolated following prior exposure to CZA. Among these, 20 distinct types of KPC variants were characterized, including KPC-33 (*n* = 11), KPC-35 (*n* = 3), KPC-44 (*n* = 2), KPC-14 (*n* = 1), KPC-31 (*n* = 1), KPC-46 (*n* = 1), KPC-53 (*n* = 1), KPC-58 (*n* = 1), KPC-71 (*n* = 1), KPC-78 (*n* = 1), KPC-90 (*n* = 1), KPC-93 (*n* = 1), KPC-144 (*n* = 1), KPC-170 (*n* = 1), and six novel KPC variants. Three of the six novel KPC variants have been documented in our prior study (12). The remaining novel variants included one strain displaying the amino acid substitution D179Y in conjunction with Y241S. Another variant had a 2-amino acid (TY) insertion between positions 179 and 180 in conjunction with substitution H273R. Finally, one displayed the amino acid substitution D179Y in conjunction with P267S. The predominant mutation sites among the identified KPC variants were located in the Ω-loop region, affecting 25 out of 33 isolates.

The carbapenem MIC and CZA MIC of all strains are shown in Table S5. A majority of the KPC variants-producing strains (27/33; 81.8%) demonstrated restored imipenem susceptibility (MIC ≤ 4 µg/mL), including strains producing KPC-14, KPC-31, KPC-33, KPC-35, KPC-46, KPC-53, KPC-71, KPC-78, KPC-90, KPC-170, and the six novel KPC variants (Table S4).

### Risk factors for development of CZA resistance in KPC-Kp strains

We further evaluated the characteristics and antibiotic exposures associated with the development of CZA resistance in KPC-Kp. Variables with a *P* value <0.1 in univariate analysis, including age, the presence of *bla*_KPC-2_, CZA use, and carbapenem use, were included in a multivariate logistic regression model (Table 3). CZA use was identified as an independent risk factor for the emergence of CZA resistance (OR = 9.47, 95% CI 2.41–37.15, *P* = 0.001), whereas carbapenem use was associated with a reduced risk (OR = 0.27, 95% CI 0.09–0.78, *P* = 0.015). A subgroup analysis was performed among 33 patients with KPC variant-producing *K. pneumoniae* and their matched controls (Table 4). The risk factors were similar: receipt of CZA was identified as an independent risk factor for the emergence of CZA resistance (OR = 17.23, 95% CI 1.85–160.87, *P* = 0.013), whereas carbapenem use was associated with a reduced risk (OR = 0.25, 95% CI 0.07–0.92, *P* = 0.037).

**TABLE 3:** Univariate and multivariate logistic regression analysis of risk factors for the *in vivo* emergence of ceftazidime-avibactam resistance in KPC-producing *Klebsiella pneumoniae*

Variable	Univariate		Multivariate	
	OR (95% CI)	*P* value	OR (95% CI)	*P* value
Age	0.98 (0.95–1.00)	0.087		
*bla*_KPC-2_	0.15 (0.02–1.28)	0.083		
Ceftazidime-avibactam use	13.39 (3.62–49.58)	<0.001	9.47 (2.41–37.15)	0.001
Carbapenem use	0.22 (0.09–0.56)	0.001	0.27 (0.09–0.78)	0.015

**TABLE 4:** Univariate and multivariate logistic regression analysis of risk factors for the *in vivo* emergence of ceftazidime-avibactam resistance in KPC variant-producing *Klebsiella pneumoniae*^*a*^

Variable	Univariate		Multivariate	
	OR (95% CI)	*P* value	OR (95% CI)	*P* value
Age	0.96 (0.93–0.99)	0.020		
Ceftazidime-avibactam use	30.12 (3.67–246.98)	0.002	17.23 (1.85–160.87)	0.013
Carbapenem use	0.19 (0.06–0.57)	0.003	0.25 (0.07–0.92)	0.037
Colistin use	0.32 (0.09–1.14)	0.079		
Anti-MRSA antibiotics use	2.38 (0.88–6.39)	0.087		

^*a*^ MRSA, methicillin-resistant *Staphylococcus aureus*.

## DISCUSSION

The present study identified prior CZA therapy as the only independent predictor of subsequent CZA resistance in KPC-Kp, especially for KPC variant-producing strains. Notably, prior carbapenem use was associated with a lower risk of *in vivo* development of CZA resistance.

The risk factors associated with the emergence of CZA resistance in KPC-Kp remain poorly defined, largely due to the limited number of CZA-resistant isolates collected in previous studies (10, 11, 20, 21). A recent review highlighted that most patients infected with CZA-resistant, KPC variant-producing *K. pneumoniae* had been previously exposed to CZA, suggesting that CZA therapy might be a risk factor for inducing mutations of *bla*_KPC_ (9). Another study found that renal replacement therapy was associated with the development of CZA resistance among patients receiving CZA treatments (20). Our study was the first case-control study to demonstrate that prior CZA use is the only independent risk factor associated with the emergence of CZA resistance in KPC-Kp. This finding underscores the importance of antimicrobial stewardship in preserving the clinical utility of CZA and in minimizing the risk of resistance development. In our study, the median duration of CZA exposure prior to the detection of resistance was 11 days (range, 2–35 days). These results highlight the need for microbiological surveillance in patients undergoing CZA treatment, particularly when the clinical condition deteriorates, to early detect the emergence of CZA resistance. Our previous study showed that most CZA-R strains carrying KPC variants had exposure to CZA (12). The current findings further showed that the use of CZA was an independent risk factor for the *in vivo* emergence of CZA-R KPC-Kp. The selective pressure imposed by CZA therapy warrants caution.

Another notable finding of this study was that prior receipt of carbapenem, including meropenem and doripenem, was independently associated with a reduced risk for the emergence of CZA resistance. This protective effect may be attributed to the restored susceptibility to carbapenem observed in a majority of KPC variant-producing isolates. Previous studies have proposed that combination therapy with CZA and carbapenems may reduce the risk of the emergence of KPC variants, given that CZA inhibits wild-type KPC enzymes, while imipenem or meropenem retains activity against many KPC variants (9, 10). Our findings also raised the possibility that other novel β-lactam/β-lactamase inhibitors recommended by U.S. guidance, e.g., MVB and IMI/REL, may represent superior options for treating KPC-producing *Enterobacterales*. Both agents included a carbapenem, which may reduce the likelihood of selecting for KPC variants compared to CZA. Nevertheless, comparative data between these agents remain limited. Only one study has directly compared MVB and CZA in the treatment of CRE infections (21). While no significant difference in clinical success was observed between treatment groups, patients receiving MVB had a lower incidence of resistance emergence than those treated with CZA (0% versus 2.9%). Taken together, these findings underscore the urgent need for comparative studies evaluating different therapeutic options for KPC-producing *K. pneumoniae*, particularly with respect to their potential to drive resistance development during treatment.

Of the 45 CZA-resistant *K. pneumoniae* isolates, 33 (73.3%) were KPC variant-producing strains. In total, 20 types of KPC variants were identified among these isolates. The most common variants were KPC-33 (*n* = 11), followed by KPC-35 (*n* = 3), KPC-44 (*n* = 2). To our knowledge, this collection represents one of the largest series of CZA-resistant, KPC-producing *K. pneumoniae* isolates reported in Asia to date. Phenotypically, 27 of the 33 KPC variant-producing strains (81.8%) exhibited restored susceptibility to imipenem, defined by an MIC ≤4 µg/mL. All KPC variant-producing isolates except those with KPC-44, KPC-58, KPC-93, or KPC-144 had restored susceptibility to imipenem. The restoration of carbapenem susceptibility in CZA-resistant strains has important clinical implications. In isolates encoding *bla*_KPC_ variants, reduced carbapenem MIC increases the likelihood that MVB or IMI/REL remain effective against these organisms (22–24). Additionally, there are case reports demonstrating successful salvage therapy with MVB in patients with infections caused by CZA-resistant *K. pneumoniae* (25, 26). The findings may support the future roles of MVB or IMI/REL in the treatment of KPC-Kp infections.

Our study has some limitations. First, it was conducted at a single center, which limited generalizability. Second, the sample size was relatively modest, which limited statistical power. Furthermore, due to the limited cases with paired KPC-Kp strains, some clinically relevant variables, such as the source of isolates, could not be matched between the cohorts. Third, due to the retrospective design, the timing of collecting cultures was decided by the physician, making it difficult to determine the precise time when CZA resistance emerged. Fourth, we did not survey other mechanisms of CZA resistance, such as KPC copy number, porin defect, or expression of efflux pump. Fifth, whole-genome sequencing was only performed on a subset of strains in this article due to resource limitations, precluding detailed analysis of clonal transmission and resistance gene contexts.

In conclusion, our study found that prior CZA use is the main risk factor for the emergence of CZA resistance, highlighting the necessity of judicious antibiotic use. Conversely, carbapenem use was associated with a protective effect for the emergence of CZA resistance, suggestive that carbapenem-based β-lactam/β-lactamase inhibitors (e.g., MVB or IMI/REL) should be further explored for their roles in clinical practice.

## Data Availability

The genome sequence of 20 pairs of CZA-R KPC-Kp and their parental CZA-S KPC-Kp strains (total 40 strains) has been deposited at NCBI under the BioSample number SAMN56997930–SAMN56997969.

## SUPPLEMENTAL MATERIAL

Fig. S1; Tables S1 to S5 spectrum.00972-26-s0001.docx Strain numbers of isolates which underwent whole-genome sequencing, detailed information of 45 cases and their matched controls, and PFGE results.

ASM does not own the copyrights to Supplemental Material that may be linked to, or accessed through, an article. The authors have granted ASM a non-exclusive, world-wide license to publish the Supplemental Material files. Please contact the corresponding author directly for reuse.
