# Supplementary material for: Risk factors for in vivo emergence of ceftazidime-avibactam resistance in KPC-producing Klebsiella pneumoniae and the associated resistance mechanisms: a case-control study
Source: Microbiol Spectr. 2026 May 4;14(6):e00972-26. doi: 10.1128/spectrum.00972-26 (PMC13227984; doi:10.1128/spectrum.00972-26)

**Supplementary Table S1.** Stain numbers of isolates which underwent whole-genome sequencing

|  | **First strain (CZA-S)** | **Second strain (CZA-R)** |
| --- | --- | --- |
| Case-2 | NCRE-1505 | NCRE-1519 |
| Case-3 | KP-3469 | NCRE-1594 |
| Case-5 | NCRE-1732 | NCRE-1849 |
| Case-6 | KP-3700 | NCRE-1883 |
| Case-7 | NCRE-1971 | NCRE-2063 |
| Case-9 | NCRE-2115 | NCRE-2165 |
| Case-10 | NCRE-2125 | NCRE-2178 |
| Case-12 | NCRE-2196 | NCRE-2225 |
| Case-13 | NCRE-2244 | NCRE-2270 |
| Case-14 | NCRE-2266 | NCRE-2277 |
| Case-15 | KP-4005 | NCRE-2330 |
| Case-16 | NCRE-2329 | NCRE-2347 |
| Case-17 | KP-4049 | NCRE-2379 |
| Case-18 | NCRE-2383 | NCRE-2411 |
| Case-19 | NCRE-2433 | CZA-440 |
| Case-20 | NCRE-2476 | NCRE-2511 |
| Case-22 | NCRE-2540 | NCRE-2579 |
| Case-23 | KP-4218 | NCRE-2583 |
| Case-25 | NCRE-2639 | NCRE-2650 |
| Case-40 | NCRE-1728 | NCRE-1836 |

**Supplementary Table S2.** Detailed information of 45 cases and their matched controls

| Group number | Case/control | First strain | | Second strain | | Days between isolation |
| --- | --- | --- | --- | --- | --- | --- |
|  |  | Infection/colonization | Culture site | Infection/colonization | Culture site |  |
| 1 | Case | Infection | Urine | Infection | Blood | 10 |
|  | Control | Infection | Respiratory system | Infection | Blood | 10 |
| 2 | Case | Infection | Respiratory system | Infection | Respiratory system | 21 |
|  | Control | Infection | Blood | Infection | Blood | 20 |
| 3 | Case | Infection | Blood | Infection | Respiratory system | 17 |
|  | Control | Infection | Respiratory system | Infection | Ascites | 17 |
| 5 | Case | Infection | Respiratory system | Infection | Respiratory system | 50 |
|  | Control | Infection | Blood | Colonization | Wound | 49 |
| 6 | Case | Infection | Blood | Colonization | Respiratory system | 5 |
|  | Control | Infection | Blood | Infection | Respiratory system | 5 |
| 7 | Case | Infection | Respiratory system | Infection | Respiratory system | 22 |
|  | Control | Colonization | Respiratory system | Infection | Respiratory system | 22 |
| 8 | Case | Infection | Respiratory system | Infection | Respiratory system | 9 |
|  | Control | Infection | Ascites | Infection | Wound (surgical site) | 9 |
| 9 | Case | Infection | Bile | Infection | Bile | 22 |
|  | Control | Infection | Urine | Infection | Blood | 22 |
| 10 | Case | Infection | Urine | Infection | Respiratory system | 25 |
|  | Control | Infection | Urine | Infection | Urine | 25 |
| 11 | Case | Infection | Blood | Infection | Bile | 56 |
|  | Control | Colonization | Urine | Infection | Respiratory system | 56 |
| 12 | Case | Infection | Respiratory system | Colonization | Respiratory system | 16 |
|  | Control | Infection | Blood | Infection | Abscess | 15 |
| 13 | Case | Infection | Bile | Infection | Respiratory system | 16 |
|  | Control | Infection | Respiratory system | Infection | Blood | 14 |
| 14 | Case | Infection | Ascites | Colonization | Ascites | 7 |
|  | Control | Infection | Blood | Infection | Respiratory system | 7 |
| 15 | Case | Infection | Blood | Infection | Respiratory system | 16 |
|  | Control | Infection | Blood | Colonization | Respiratory system | 16 |
| 16 | Case | Infection | Respiratory system | Colonization | Respiratory system | 14 |
|  | Control | Infection | Wound (surgical site) | Infection | Abscess in abdomen | 14 |
| 17 | Case | Infection | Blood | Colonization | Bile | 17 |
|  | Control | Infection | Urine | Infection | Respiratory system | 19 |
| 18 | Case | Infection | Urine | Colonization | Urine | 20 |
|  | Control | Infection | Wound (bedsore) | Infection | Wound (bedsore) | 20 |
| 19 | Case | Infection | Urine | Infection | Urine | 6 |
|  | Control | Infection | Blood | Infection | Ascites | 6 |
| 20 | Case | Infection | Urine | Infection | Urine | 27 |
|  | Control | Colonization | Urine | Colonization | Urine | 25 |
| 21 | Case | Infection | Urine | Infection | Urine | 35 |
|  | Control | Infection | Respiratory system | Infection | Respiratory system | 35 |
| 22 | Case | Infection | Urine | Infection | Respiratory system | 41 |
|  | Control | Infection | Respiratory system | Colonization | Respiratory system | 40 |
| 23 | Case | Infection | Blood | Infection | Urine | 39 |
|  | Control | Colonization | Urine | Infection | Urine | 41 |
| 24 | Case | Infection | Blood | Colonization | Respiratory system | 12 |
|  | Control | Infection | Respiratory system | Infection | Respiratory system | 11 |
| 25 | Case | Infection | Bile | Infection | Bile | 12 |
|  | Control | Infection | Blood | Infection | Blood | 12 |
| 26 | Case | Infection | Blood | Infection | Respiratory system | 21 |
|  | Control | Infection | Respiratory system | Infection | Respiratory system | 21 |
| 27 | Case | Infection | Respiratory system | Colonization | Respiratory system | 17 |
|  | Control | Infection | Blood | Infection | Respiratory system | 16 |
| 28 | Case | Infection | Respiratory system | Infection | Respiratory system | 21 |
|  | Control | Infection | Ascites | Colonization | Wound | 21 |
| 29 | Case | Infection | Respiratory system | Colonization | Respiratory system | 12 |
|  | Control | Colonization | Respiratory system | Colonization | Respiratory system | 14 |
| 30 | Case | Infection | Respiratory system | Infection | Respiratory system | 31 |
|  | Control | Infection | Respiratory system | Infection | Respiratory system | 30 |
| 31 | Case | Infection | Respiratory system | Infection | Respiratory system | 14 |
|  | Control | Infection | Urine | Infection | Urine | 14 |
| 32 | Case | Infection | Ascites | Infection | Ascites | 9 |
|  | Control | Infection | Blood | Infection | Bile | 9 |
| 33 | Case | Infection | Blood | Colonization | Urine | 39 |
|  | Control | Infection | Respiratory system | Infection | Blood | 40 |
| 34 | Case | Infection | Wound (jejunostomy) | Infection | Respiratory system | 26 |
|  | Control | Infection | Respiratory system | Infection | Respiratory system | 24 |
| 35 | Case | Colonization | Respiratory system | Infection | Respiratory system | 55 |
|  | Control | Colonization | Urine | Infection | Respiratory system | 53 |
| 36 | Case | Infection | Respiratory system | Infection | Respiratory system | 19 |
|  | Control | Infection | Blood | Infection | Urine | 18 |
| 37 | Case | Infection | Blood | Infection | Blood | 12 |
|  | Control | Infection | Blood | Infection | Respiratory system | 13 |
| 38 | Case | Infection | Blood | Infection | Blood | 8 |
|  | Control | Infection | Respiratory system | Infection | Respiratory system | 8 |
| 39 | Case | Infection | Bile | Infection | Bile | 19 |
|  | Control | Infection | Blood | Infection | Pleural fluid | 19 |
| 40 | Case | Infection | Respiratory system | Infection | Respiratory system | 43 |
|  | Control | Infection | Respiratory system | Infection | Blood | 43 |
| 42 | Case | Infection | Urine | Infection | Urine | 29 |
|  | Control | Colonization | Urine | Infection | Respiratory system | 29 |
| 43 | Case | Infection | Blood | Infection | Urine | 42 |
|  | Control | Infection | Blood | Infection | Blood | 42 |
| 44 | Case | Infection | Respiratory system | Colonization | Urine | 46 |
|  | Control | Infection | Respiratory system | Infection | Respiratory system | 45 |
| 47 | Case | Infection | Wound (bedsore) | Colonization | Wound (bedsore) | 20 |
|  | Control | Infection | Bile | Infection | Bile | 20 |
| 48 | Case | Infection | Respiratory system | Infection | Urine | 46 |
|  | Control | Infection | Blood | Infection | Blood | 46 |
| 49 | Case | Infection | Urine | Infection | Urine | 31 |
|  | Control | Infection | Respiratory system | Colonization | Respiratory system | 31 |

Abbreviations: CZA, ceftazidime-avibactam

**Supplementary Table S3. Dosage and duration of ceftazidime-avibactam**

| Group number | Case/control | Dosage | Days of ceftazidime-avibactam use |
| --- | --- | --- | --- |
|  |  |  |  |
| 1 | Case | N/A | 0 |
|  | Control | N/A | 0 |
| 2 | Case | 940mg every 2 days | 2 |
|  | Control | 2500mg every 8 hours | 5 |
| 3 | Case | 940mg every 2 days | 11 |
|  | Control | 2500mg every 8 hours | 6 |
| 5 | Case | 940mg every 2 days | 15 |
|  | Control | 1250mg every 8 hours | 15 |
| 6 | Case | 1250mg every 8 hours | 4 |
|  | Control | 2500mg every 8 hours | 4 |
| 7 | Case | 2500mg every 8 hours | 12 |
|  | Control | N/A | 0 |
| 8 | Case | 2500mg every 8 hours | 8 |
|  | Control | 940mg every 2 days | 7 |
| 9 | Case | 940mg every 12 hours | 14 |
|  | Control | 2500mg every 8 hours | 5 |
| 10 | Case | 940mg every 12 hours | 9 |
|  | Control | 2500mg every 8 hours | 8 |
| 11 | Case | 2500mg every 8 hours | 17 |
|  | Control | N/A | 0 |
| 12 | Case | 940mg every 2 days | 11 |
|  | Control | 940mg every 2 days | 14 |
| 13 | Case | 2500mg every 8 hours | 13 |
|  | Control | N/A | 0 |
| 14 | Case | 940mg every 2 days | 7 |
|  | Control | 2500mg every 8 hours | 2 |
| 15 | Case | 1250mg every 8 hours | 7 |
|  | Control | N/A | 0 |
| 16 | Case | 1250mg every 8 hours | 11 |
|  | Control | 940mg every 2 days | 7 |
| 17 | Case | 940mg every 12 hours | 11 |
|  | Control | 2500mg every 8 hours | 8 |
| 18 | Case | 1250mg every 8 hours | 6 |
|  | Control | N/A | 0 |
| 19 | Case | N/A | 0 |
|  | Control | 1250mg every 8 hours | 5 |
| 20 | Case | 940mg every 24 hours | 13 |
|  | Control | N/A | 0 |
| 21 | Case | 940mg every 12 hours | 10 |
|  | Control | N/A | 0 |
| 22 | Case | 2500mg every 8 hours | 22 |
|  | Control | 2500mg every 8 hours | 11 |
| 23 | Case | 1250mg every 8 hours | 26 |
|  | Control | N/A | 0 |
| 24 | Case | 940mg every 2 days | 8 |
|  | Control | 940mg every 2 days | 2 |
| 25 | Case | 2500mg every 8 hours | 11 |
|  | Control | N/A | 0 |
| 26 | Case | 2500mg every 8 hours | 14 |
|  | Control | 2500mg every 8 hours | 13 |
| 27 | Case | 940mg every 2 days | 11 |
|  | Control | N/A | 0 |
| 28 | Case | 940mg every 2 days | 14 |
|  | Control | 2500mg every 8 hours | 12 |
| 29 | Case | 2500mg every 8 hours | 8 |
|  | Control | N/A | 0 |
| 30 | Case | 1250mg every 8 hours | 12 |
|  | Control | N/A | 0 |
| 31 | Case | 940mg every 2 days | 11 |
|  | Control | 1250mg every 8 hours | 9 |
| 32 | Case | 940mg every 12 hours | 6 |
|  | Control | 2500mg every 8 hours | 6 |
| 33 | Case | 940mg every 2 days | 35 |
|  | Control | N/A | 0 |
| 34 | Case | 2500mg every 8 hours | 17 |
|  | Control | N/A | 0 |
| 35 | Case | 2500mg every 8 hours | 3 |
|  | Control | N/A | 0 |
| 36 | Case | 2500mg every 8 hours | 15 |
|  | Control | 940mg every 2 days | 4 |
| 37 | Case | 940mg every 12 hours | 12 |
|  | Control | N/A | 0 |
| 38 | Case | 940mg every 2 days | 7 |
|  | Control | N/A | 0 |
| 39 | Case | 940mg every 2 days | 10 |
|  | Control | 1250mg every 8 hours | 11 |
| 40 | Case | 1250mg every 8 hours | 7 |
|  | Control | 940mg every 2 days | 9 |
| 42 | Case | 2500mg every 8 hours | 12 |
|  | Control | N/A | 0 |
| 43 | Case | 2500mg every 8 hours | 33 |
|  | Control | N/A | 0 |
| 44 | Case | 940mg every 2 days | 11 |
|  | Control | 1250mg every 8 hours | 15 |
| 47 | Case | 2500mg every 8 hours | 17 |
|  | Control | 940mg every 2 days | 10 |
| 48 | Case | 940mg every 12 hours | 21 |
|  | Control | 2500mg every 8 hours | 30 |
| 49 | Case | 2500mg every 8 hours | 8 |
|  | Control | 940mg every 24 hours | 11 |

N/A: not applicable

**Supplementary Table S4.** Genotype of ceftazidime-avibactam-resistant KPC-producing *Klebsiella pneumoniae* strains in this study

| Strains | KPC | | MIC (mg/L) | |
| --- | --- | --- | --- | --- |
|  | *bla*_KPC_ mutations | variant | CZA | IPM |
| Case-1 | wild type | KPC-2 | 16 | ≥16 |
| Case-2 | KPC-2_V277_dupYTRAPNKDDKHSEAV_I278 | KPC-44 | 128 | ≥16 |
| Case-3 | KPC-2_179_insTY_180 | KPC-90 | >256 | 2 |
| Case-5 | wild type | KPC-3 | 16 | ≥16 |
| Case-6 | KPC-2_D271_insNRAPNKDD_K272 | KPC-58 | 128 | ≥16 |
| Case-7 | KPC-2_D179Y | KPC-33 | 128 | 1 |
| Case-8 | KPC-2_D179Y | KPC-33 | 64 | 2 |
| Case-9 | wild type | KPC-3 | 32 | ≥16 |
| Case-10 | KPC-2_wild type plus D179Y | KPC-2 plus KPC-33 | 128 | ≥16 |
| Case-11 | KPC-3_D179Y | KPC-31 | 128 | 1 |
| Case-12 | KPC-2_L169P | KPC-35 | 16 | 1 |
| Case-13 | KPC-3_L169P | KPC-46 | 64 | 0.5 |
| Case-14 | KPC-2_D179Y | KPC-33 | >256 | 1 |
| Case-15 | KPC-2_D179N | KPC-170 | 64 | 2 |
| Case-16 | KPC-2_D179Y plus T264S | new variant | 64 | ≤0.25 |
| Case-17 | wild type | KPC-2 | 16 | ≥16 |
| Case-18 | KPC-3_L169_dupEL_N170 | KPC-53 | 16 | 0.5 |
| Case-19 | wild type | KPC-2 | 32 | ≥16 |
| Case-20 | wild type | KPC-2 | 32 | ≥16 |
| Case-21 | KPC-2_D179Y | KPC-33 | >256 | 1 |
| Case-22 | KPC-2_L169P | KPC-35 | >256 | 0.5 |
| Case-23 | KPC-2_N268_ins_NRAPN_K269 | KPC-93 | >256 | ≥16 |
| Case-24 | KPC-2_D179Y | KPC-33 | >256 | ≤0.25 |
| Case-25 | KPC-2_A172T | KPC-144 | 32 | ≥16 |
| Case-26 | KPC-2_L169P | KPC-35 | 32 | 1 |
| Case-27 | wild type | KPC-2 | 32 | ≥16 |
| Case-28 | KPC-2_D179Y | KPC-33 | 256 | 0.5 |
| Case-29 | KPC-2_ Y241_delGT_A244 | KPC-14 | >256 | ≤0.25 |
| Case-30 | KPC-2_D179Y | KPC-33 | 128 | ≤0.25 |
| Case-31 | KPC-2_D179A plus Y241S | new variant | ≥16 | 1 |
| Case-32 | KPC-2_D179A | KPC-78 | 64 | ≤0.25 |
| Case-33 | KPC-2_D179Y | KPC-33 | >256 | ≤0.25 |
| Case-34 | KPC-2_V277_dupYTRAPNKDDKHSEAV_I278 | KPC-44 | 128 | 8 |
| Case-35 | wild type | KPC-2 | 16 | ≥16 |
| Case-36 | KPC-2_T180_insS_S181 | KPC-71 | 128 | ≤0.25 |
| Case-37 | wild type | KPC-2 | 32 | ≥16 |
| Case-38 | KPC-2_T180_insS_S181 | new variant | 128 | 0.75 |
| Case-39 | KPC-2_S182_insS_P183 | new variant | 64 | 4 |
| Case-40 | wild type | KPC-3 | 32 | ≥16 |
| Case-42 | wild type | KPC-2 | 16 | ≥16 |
| Case-43 | KPC-2_D179Y | KPC-33 | 256 | 1 |
| Case-44 | wild type | KPC-2 | 32 | ≥16 |
| Case-47 | KPC-2_D179_insTY_T180 plus H273R | new variant | >256 | 2 |
| Case-48 | KPC-2_D179Y | KPC-33 | 128 | 0.5 |
| Case-49 | KPC-2_D179Y & P267S | new variant | >256 | ≤0.25 |

Abbreviations: CZA, ceftazidime-avibactam; IPM, imipenem

**Supplementary Table S5. Carbapenem minimum inhibitory concentration (MIC) and ceftazidime-avibactam MIC of all KPC-producing Klebsiella pneumoniae strains**

| Group number | Case/control | First strain | | Second strain | |
| --- | --- | --- | --- | --- | --- |
|  |  | CZA MIC (mg/L) | IPM MIC (mg/L) | CZA MIC (mg/L) | IPM MIC (mg/L) |
| 1 | Case | 2 | ≥16 | 16 | ≥16 |
|  | Control | 2 | ≥16 | 4 | ≥16 |
| 2 | Case | 8 | ≥16 | 128 | ≥16 |
|  | Control | 8 | ≥16 | 8 | ≥16 |
| 3 | Case | 2 | ≥16 | >256 | 2 |
|  | Control | 1 | ≥16 | 1 | ≥16 |
| 5 | Case | 8 | ≥16 | 16 | ≥16 |
|  | Control | 2 | ≥16 | 2 | ≥16 |
| 6 | Case | 2 | ≥16 | 128 | ≥16 |
|  | Control | 4 | ≥16 | 4 | ≥16 |
| 7 | Case | 2 | ≥16 | 128 | 1 |
|  | Control | 4 | ≥16 | 1.5 | ≥16 |
| 8 | Case | 2 | ≥16 | 64 | 2 |
|  | Control | 2 | ≥16 | 8 | ≥16 |
| 9 | Case | 8 | ≥16 | 32 | ≥16 |
|  | Control | 8 | ≥16 | 4 | ≥16 |
| 10 | Case | 2 | ≥16 | 128 | ≥16 |
|  | Control | 1 | ≥16 | 1 | ≥16 |
| 11 | Case | 2 | ≥16 | 128 | 1 |
|  | Control | 2 | ≥16 | 4 | ≥16 |
| 12 | Case | 2 | ≥16 | 16 | 1 |
|  | Control | 1 | ≥16 | 1 | ≥16 |
| 13 | Case | 8 | ≥16 | 64 | 0.5 |
|  | Control | 2 | ≥16 | 2 | ≥16 |
| 14 | Case | 8 | ≥16 | >256 | 1 |
|  | Control | 2 | ≥16 | 4 | ≥16 |
| 15 | Case | 2 | ≥16 | 64 | 2 |
|  | Control | 4 | ≥16 | 4 | ≥16 |
| 16 | Case | 0.75 | ≥16 | 64 | ≤0.25 |
|  | Control | 4 | ≥16 | 4 | ≥16 |
| 17 | Case | 4 | ≥16 | 16 | ≥16 |
|  | Control | 2 | ≥16 | 1 | ≥16 |
| 18 | Case | 8 | ≥16 | 16 | 0.5 |
|  | Control | 1 | ≥16 | ≤0.5 | ≥16 |
| 19 | Case | 4 | ≥16 | 32 | ≥16 |
|  | Control | 4 | ≥16 | 2 | ≥16 |
| 20 | Case | 4 | ≥16 | 32 | ≥16 |
|  | Control | 2 | ≥16 | 8 | ≥16 |
| 21 | Case | 4 | ≥16 | >256 | 1 |
|  | Control | 2 | ≥16 | 2 | ≥16 |
| 22 | Case | 1 | ≥16 | >256 | 0.5 |
|  | Control | 1 | ≥16 | 1 | ≥16 |
| 23 | Case | 4 | ≥16 | >256 | ≥16 |
|  | Control | 2 | ≥16 | 1 | ≥16 |
| 24 | Case | 3 | ≥16 | >256 | ≤0.25 |
|  | Control | 1 | ≥16 | 2 | ≥16 |
| 25 | Case | 1 | ≥16 | 32 | ≥16 |
|  | Control | 4 | ≥16 | 4 | ≥16 |
| 26 | Case | 1 | ≥16 | 32 | 1 |
|  | Control | 2 | ≥16 | 4 | ≥16 |
| 27 | Case | 4 | ≥16 | 32 | ≥16 |
|  | Control | 1 | ≥16 | 1 | ≥16 |
| 28 | Case | 4 | ≥16 | 256 | 0.5 |
|  | Control | 4 | ≥16 | 2 | ≥16 |
| 29 | Case | 2 | ≥16 | >256 | ≤0.25 |
|  | Control | 2 | ≥16 | 2 | ≥16 |
| 30 | Case | 2 | ≥16 | 128 | ≤0.25 |
|  | Control | 4 | ≥16 | 4 | ≥16 |
| 31 | Case | 1 | ≥16 | ≥16 | 1 |
|  | Control | 1.5 | ≥16 | 4 | ≥16 |
| 32 | Case | 4 | ≥16 | 64 | ≤0.25 |
|  | Control | 4 | ≥16 | 4 | ≥16 |
| 33 | Case | 4 | ≥16 | >256 | ≤0.25 |
|  | Control | 2 | ≥16 | 2 | ≥16 |
| 34 | Case | 1 | ≥16 | 128 | 8 |
|  | Control | 4 | ≥16 | 4 | ≥16 |
| 35 | Case | 1 | ≥16 | 16 | ≥16 |
|  | Control | 2 | ≥16 | 2 | ≥16 |
| 36 | Case | 1 | ≥16 | 128 | ≤0.25 |
|  | Control | 2 | ≥16 | 2 | ≥16 |
| 37 | Case | 4 | ≥16 | 32 | ≥16 |
|  | Control | 8 | ≥16 | 8 | ≥16 |
| 38 | Case | 4 | ≥16 | 128 | 0.75 |
|  | Control | 2 | ≥16 | 2 | ≥16 |
| 39 | Case | 4 | ≥16 | 64 | 4 |
|  | Control | 2 | ≥16 | 2 | ≥16 |
| 40 | Case | 8 | ≥16 | 32 | ≥16 |
|  | Control | 1.5 | ≥16 | 4 | ≥16 |
| 42 | Case | 1 | ≥16 | 16 | ≥16 |
|  | Control | 4 | ≥16 | 1 | ≥16 |
| 43 | Case | 4 | ≥16 | 256 | 1 |
|  | Control | 4 | ≥16 | 4 | ≥16 |
| 44 | Case | 1 | ≥16 | 32 | ≥16 |
|  | Control | 3 | ≥16 | 8 | ≥16 |
| 47 | Case | 4 | ≥16 | >256 | 2 |
|  | Control | 1 | ≥16 | 0.5 | ≥16 |
| 48 | Case | 1 | ≥16 | 128 | 0.5 |
|  | Control | 4 | ≥16 | 4 | ≥16 |
| 49 | Case | 4 | ≥16 | >256 | ≤0.25 |
|  | Control | 1.5 | ≥16 | 2 | ≥16 |

Abbreviations: CZA, ceftazidime-avibactam; IPM, imipenem

**Supplementary Figure 1.** PFGE of 26 paired ceftazidime-avibactam-resistant and ceftazidime-avibactam-susceptible strains were performed. Thirteen paired strains showed indistinguishable pattern, and ≤ 3 different fragments were observed in 12 paired strains. One paired strain (case-46) showed >3 different fragments.


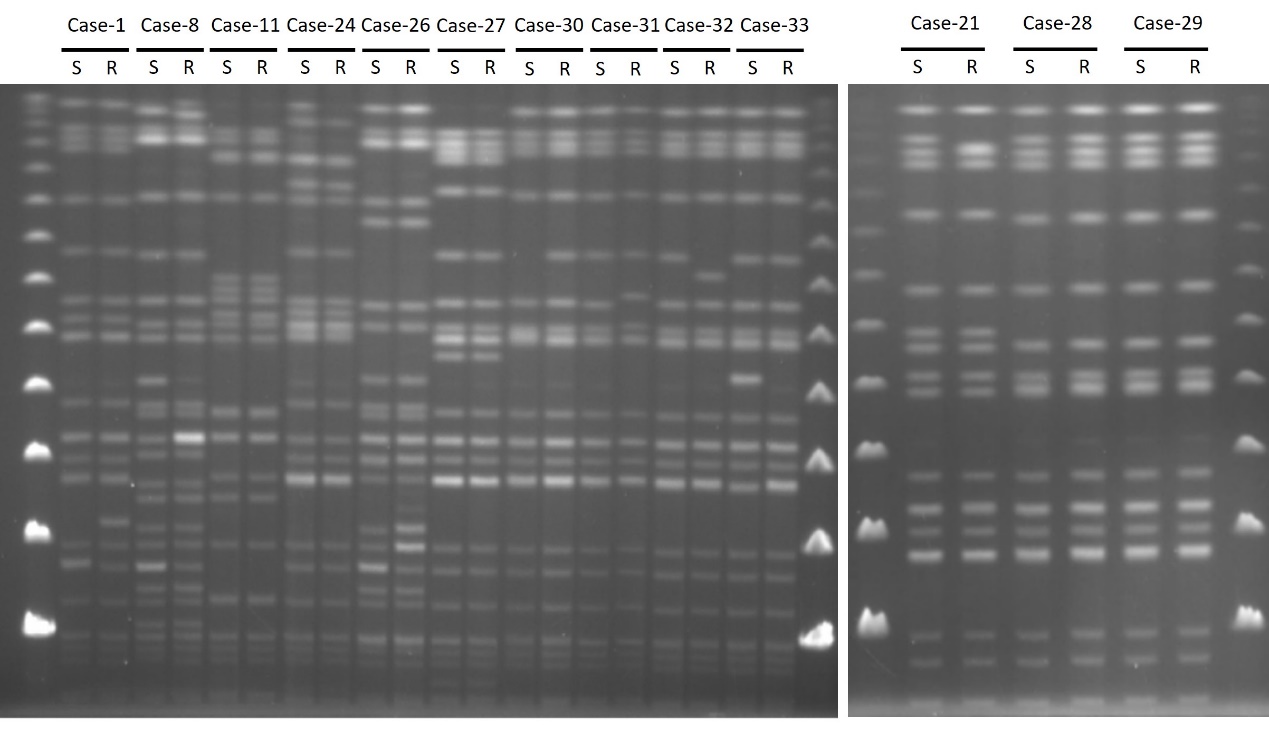


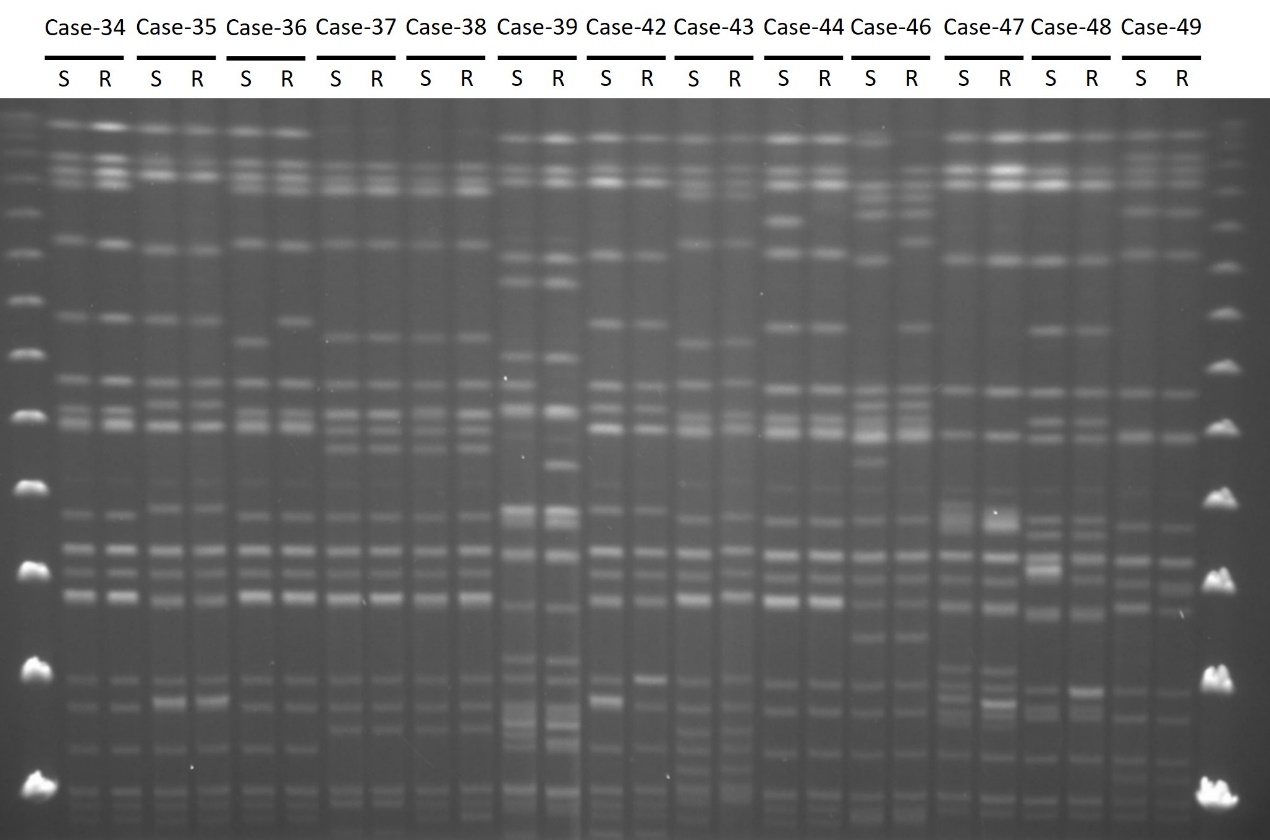

Supplement: Fig. S1; Tables S1 to S5 — Strain numbers of isolates which underwent whole-genome sequencing, detailed information of 45 cases and their matched controls, and PFGE results. [file spectrum.00972-26-s0001.docx]
